# Using the properties of the odds ratio to improve precision in meta-analysis: an update on the benefits of targeted deployment of physician-led interprofessional pre-hospital teams on the care of critically ill and injured patients

**DOI:** 10.1186/s13049-025-01345-y

**Published:** 2025-03-03

**Authors:** Ryan D. McHenry

**Affiliations:** Scottish Ambulance Service, Paisley, UK

The recent systematic review and meta-analysis titled “the benefits of targeted deployment of physician-led interprofessional pre-hospital teams on the care of critically Ill and injured patients” by Lavery and colleagues provides a useful review of the literature in an important research question [1]. The study also provides opportunity to refine its methods, and improve precision of the estimate of effect sizes, by utilising the symmetrical properties of the odds ratio. Given that the odds ratio for an outcome is the inverse of the odds ratio for other, mutually exclusive, outcomes [2], a simple inversion of mortality outcome effects will produce survival outcome effects, and allow pooling of all the outcomes reported in these studies.

This analysis was undertaken using the same random-effects pooling methodology as the original work with the meta package in R (R Foundation for Statistical Computing, Vienna, Austria) [3] and produces similar effect sizes with the benefits of increased precision, and potentially the improved interpretability of a single result (Fig. 1).

**Fig. 1:**
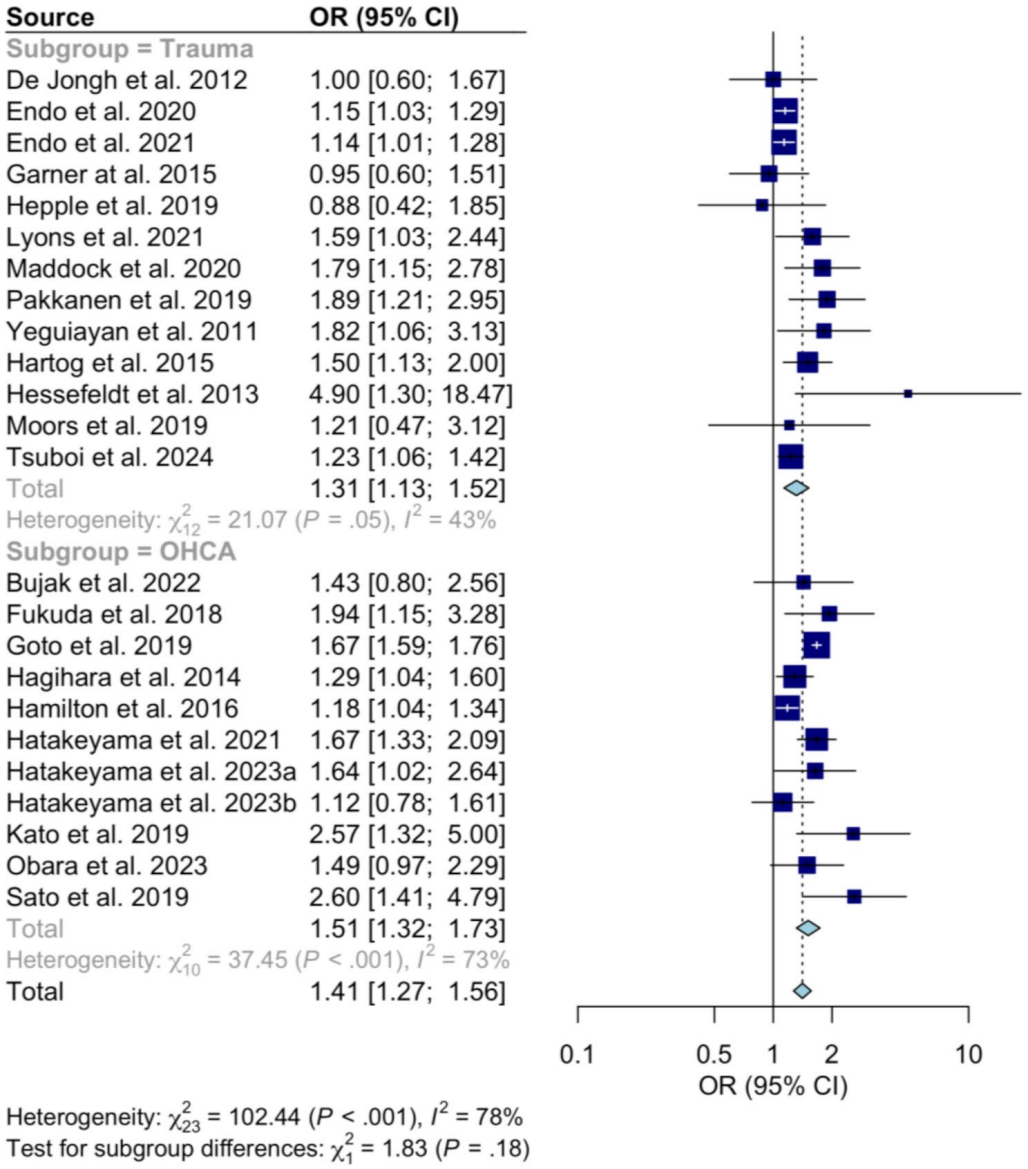
A forest plot showing the survival outcomes in patients receiving physician-based care compared to standard care

The opportunity was also taken to construct a funnel plot, which has some utility in the assessment of publication bias [4], which demonstrated visual symmetry, providing some reassurance that significant publication bias is unlikely (Fig. 2).

**Fig. 2:**
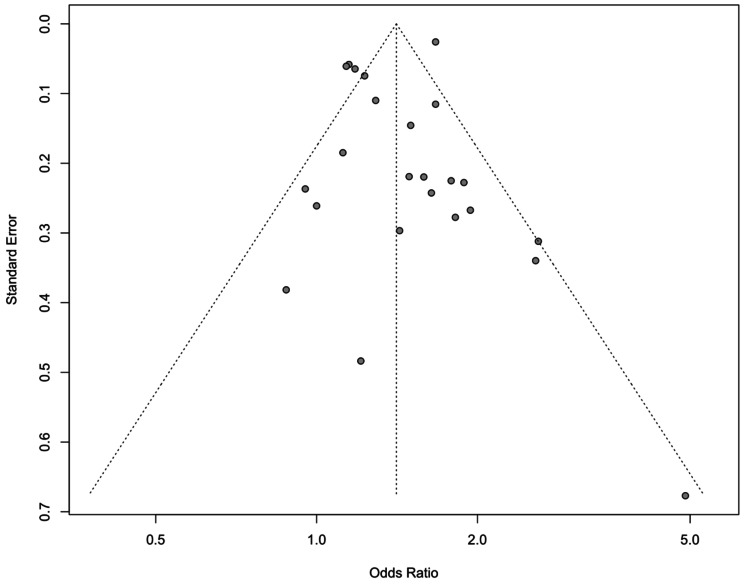
A funnel plot for studies assessing outcomes in patients receiving physician-based care compared to standard care

While this re-analysis does not mitigate the limitations of the initial study in terms of significant heterogeneity of study characteristics and outcome, it does provide a more efficient use of the available data. Improved understanding of the properties of commonly used statistical techniques is likely to result in more reliable results and greater interpretability of the available evidence.

## Data Availability

No datasets were generated or analysed during the current study.
